# Effect of breathwork on stress and mental health: A meta-analysis of randomised-controlled trials

**DOI:** 10.1038/s41598-022-27247-y

**Published:** 2023-01-09

**Authors:** Guy William Fincham, Clara Strauss, Jesus Montero-Marin, Kate Cavanagh

**Affiliations:** 1 Department of Psychology, University of Sussex, Brighton, UK; 2 Research and Development Department, Sussex Partnership NHS Foundation Trust, Brighton, UK; 3 Department of Psychiatry, Warneford Hospital, University of Oxford, Oxford, UK; 4 Teaching, Research and Innovation Unit, Parc Sanitari Sant Joan de Déu, Barcelona, Spain; 5 Consortium for Biomedical Research in Epidemiology and Public Health (CIBER Epidemiology and Public Health—CIBERESP), Madrid, Spain

**Keywords:** Psychology, Quality of life

## Abstract

Deliberate control of the breath (breathwork) has recently received an unprecedented surge in public interest and breathing techniques have therapeutic potential to improve mental health. Our meta-analysis primarily aimed to evaluate the efficacy of breathwork through examining whether, and to what extent, breathwork interventions were associated with lower levels of self-reported/subjective stress compared to non-breathwork controls. We searched PsycInfo, PubMed, ProQuest, Scopus, Web of Science, ClinicalTrials.gov and ISRCTN up to February 2022, initially identifying 1325 results. The primary outcome self-reported/subjective stress included 12 randomised-controlled trials (*k* = 12) with a total of 785 adult participants. Most studies were deemed as being at moderate risk of bias. The random-effects analysis yielded a significant small-to-medium mean effect size, *g* = − 0.35 [95% CI − 0.55, − 0.14], *z* = 3.32, *p* = 0.0009, showing breathwork was associated with lower levels of stress than control conditions. Heterogeneity was intermediate and approaching significance, *χ*^2^_11_ = 19, *p* = 0.06, *I*^2^ = 42%. Meta-analyses for secondary outcomes of self-reported/subjective anxiety (*k* = 20) and depressive symptoms (*k* = 18) showed similar significant effect sizes: *g* = − 0.32, *p* < 0.0001, and *g* = − 0.40, *p* < 0.0001, respectively. Heterogeneity was moderate and significant for both. Overall, results showed that breathwork may be effective for improving stress and mental health. However, we urge caution and advocate for nuanced research approaches with low risk-of-bias study designs to avoid a miscalibration between hype and evidence.

## Introduction

Breathwork comprises various practices which encompass regulating the way that one breathes, particularly in order to promote mental, emotional and physical health (Oxford English Dictionary)^1^. These techniques have emerged worldwide with complex historical roots from various traditions such as yoga (i.e., alternate nostril breathing) and Tibetan Buddhism (i.e., vase breathing) along with psychedelic communities (i.e., conscious connected breathing) and scientific/medical researchers and practitioners (i.e., coherent/resonant frequency breathing). Recently, breathwork has been garnering public attention and popularity in the West due to supposed beneficial effects on health and well-being^2^ in addition to the breathing-related pathology of covid-19, however it has only been partly investigated by clinical research and psychiatric medical communities.

Slow-paced breathing practices have gained most research attention thus far. Several psychophysiological mechanisms of action are proposed to underpin such techniques: from polyvagal theory and interoception literature^3^ along with enteroception, central nervous system effects, and increasing heart-rate variability (HRV) via modulation of the autonomic nervous system (ANS) and increased parasympathetic activity^4^. ANS activity can be measured using HRV, the oscillations in heart rate connected to breathing (i.e., the fluctuation in the interval between successive heart beats)^5^. Fundamentally, as one inhales and exhales, heart rate increases and decreases, respectively. Higher HRV, arising from respiratory sinus arrhythmia^6^, is typically beneficial as it translates into robust responses to changes in breathing and thus a more resilient stress-response system^7^.

Stress-response dysfunction, associated with impaired ANS activity, and low HRV are common in stress, anxiety, and depression^8–12^. This may explain why techniques like HRV biofeedback can be helpful^13^, however, it is possible that simply pacing respiration slowly at approximately 5–6 breaths/minute, requiring no monitoring equipment, can elicit similar effects^14^. Polyvagal Theory^3^, for instance, posits that vagal nerves are major channels for bidirectional communication between body and brain. Bodily feedback has profound effects on mental states as 80% of vagus nerve fibres transmit messages from body to brain^15^. Further, the neurovisceral integration model states that high vagal tone is associated with improved health along with emotional and cognitive functioning^16,17^. Vagal nerves form the main pathway of the parasympathetic nervous system, and high HRV indicates greater parasympathetic activity^7^.

Modifying breathing alters communication sent from the respiratory system, rapidly influencing brain regions regulating behaviour, thought and emotion^18^. Likewise, respiration may entrain brain electrical activity^19^, with slow breathing resulting in synchrony of brain waves^20^, thereby enabling diverse brain regions to communicate more effectively^21^. It has been observed that adept long-term Buddhist meditation practitioners can achieve states where brain waves are synchronised continuously^22^.

### Breathwork and stress

Stress, anxiety and depression have markedly exceeded pre-covid-19 pandemic population norms^23^. Thus, research is needed to address how this can be mitigated^24^. A recent survey based on more than 150,000 interviews in over 100 countries suggested that 40% of adults had experienced stress the day preceding the survey (Gallup, US)^25^. Prior to the pandemic, mental health difficulties were already a significant issue. For instance, stress has been identified by the World Health Organisation as contributing to several non-communicable diseases^26^ and a 2014 survey, led in collaboration with Harvard, of over 115 million adults showed that 72% and 60% frequently experienced financial and occupational stress, respectively (Robert Wood Johnson Foundation, US)^27^.

Chronic stress is associated with, and can significantly contribute to, many physical and mental health conditions, from hypertension and cardiovascular disease to anxiety and depression^28^. For common mental health problems such as anxiety and depression, cognitive behavioural therapy (CBT) is widely recommended in treatment guidelines worldwide^29,30^, yet many do not recover and waiting times can be long^31,32^, in addition to extensive professional training and ongoing supervision being required for therapists. Moreover, such treatment is typically individualised and offered on a one-to-one basis making it resource intensive. The present state of global mental health coupled with the access barriers to psychological therapies requires interventions that are easily accessible and scalable^7^, and manualised practices such as breathwork may meet this remit.

Breathing exercises can be easily taught to both trainers and practitioners, and learned in group settings, increasingly via synchronous and asynchronous methods remotely/online. Therefore, given the need for effective treatments that can be offered at scale with limited resources, interventions focusing on deliberately changing breathing might have significant potential. Indeed, some government public health platforms already recommend deep breathing for stress, anxiety and panic symptoms (NHS and IAPT, UK)^33,34^. However, the evidence underlying this recommendation has not been scrutinised in a comprehensive systematic review and meta-analysis and this is the aim of the current study.

Moreover, it is not only slow-paced breathing which may help reduce stress. Fast-paced breathwork may also offer therapeutic benefit as temporary voluntarily induced stress is also known to be beneficial for health and stress resilience. For example, regular physical exercise can improve stress, anxiety and depression levels^35^, along with HRV^36^. Similarly, fast-paced breathing techniques can induce short-term stress that may improve mental health^37^, and have also been shown to volitionally influence the ANS, promoting sympathetic activity^38^. There are countless breathwork techniques—and such variation in their potential modalities and underlying principles warrants exploration.

### Review aims

It is important that hype around breathwork is grounded in evidence for efficacy—and effects are not overstated to the public. Whilst some previous reviews of breathwork have been published, it is not possible to conclude the effectiveness of breathwork for stress (nor mental health in general) based on previous meta-analyses, since they have been restricted by certain factors. These include focusing on populations with impaired breathing (i.e., chronic obstructive pulmonary disease—COPD, and Asthma)^39,40^, insufficient focus on the breathwork intervention itself (i.e., including interventions where breathwork is combined with several other intervention components)^41^ making it hard to elicit separate effects, along with spanning more literature on self-reported/subjective anxiety and depression compared to stress^14^. On the other hand, systematic reviews with narrative syntheses of quantitative data may have overlooked key studies because of too much focus on a specific technique (i.e., slow breathing or diaphragmatic breathing)^4,42^, an absence of randomised-controlled trials (RCTs), scanter literature on self-reported/subjective stress compared to self-reported/subjective symptoms of anxiety and depression, along with limited databases^4^, or exclusion of unpublished studies and grey literature (i.e., theses/dissertations)^43^.

Furthermore, in keeping with the participant, intervention, control, outcome and study design (PICOS) framework^44^, there is an absence of examining dose–response correlates with effects and subgroup analyses evaluating differential effects of different breathwork interventions and how they were delivered, what controls were used, effects on populations with differing health statuses and, finally, the psychological outcome measures used. All of these are crucial for an adequate ethical, precautional and practical implementation of breathwork interventions. Accordingly, subgroup analyses were explored to account for these, for the primary outcome of stress. It could be relevant to investigate potential sources of heterogeneity in terms of effects on stress, and this might be related to how some subgroups (such as mental/physical health populations, along with nonclinical/general populations) receive the intervention. Moreover, other subgroups such as the type of breathwork intervention (i.e., slow/fast) and how it is delivered (i.e., online/in-person or individual/group-based), along with the type of comparator (active/inactive control) and outcome measure (questionnaire) used to self-report on stress, may be sources of heterogeneity and thus warrant investigation.

So far, there is no existing meta-analysis of RCTs on the effect of breathwork on psychological stress. Thus, to fill this research gap, the aim of our meta-analysis was to estimate the effect of breathwork in targeting stress. Because prolonged stress can significantly contribute to anxiety and depressive symptoms and there is considerable overlap between them^45,46^, we included these two common mental health issues as secondary outcomes, to provide a bigger picture and greater context around the findings on stress. The primary outcome was pre-registered as stress since it is a transdiagnostic variable, relevant in a variety of disorders, and also in people without a diagnosis but suffering from high levels of psychological distress^47^. This makes stress a very interesting target for breathwork-based interventions.

In brief, our research question was the following: do breathwork interventions lead to lower self-reported/subjective stress (primary outcome), anxiety, and depression (secondary outcomes) in comparison to non-breathwork control conditions? We propose this work as a first comprehensive systematic review and meta-analysis exploring the effects of breathwork on stress and mental health, to help lay a solid foundation for the field to grow and evolve in an evidence-based manner.

## Methods

We focused solely on RCTs reporting psychological measures, to gauge any potential efficacy or effectiveness of breathwork. We also explored sub-analyses for stress outcomes depending on the health status of the study population, technique, and delivery of breathwork, along with types of control groups and stress outcome measures used. Finally, we examined dose–response effects of breathwork on stress.

### Pre-registration and search strategy

Our meta-analysis was pre-registered on the international prospective register of systematic reviews PROSPERO (2022 CRD42022296709). Preferred Reporting Items for Systematic Reviews and Meta-Analyses (PRISMA) standards were applied throughout. We searched published, unpublished, and grey literature in the following five databases: PsycInfo, PubMed, ProQuest, Scopus, and Web of Science, along with two clinical trial registers: ClinicalTrials.gov and ISRCTN. The search was run up to February 2022 for all seven electronic repositories, with no date restrictions, in line with the search criteria pre-registered on Prospero, including keywords such as: breath*, respir*, random*, RCT, and stress (see Online Appendix A for the detailed search). For purposes of feasibility in conducting the search, we maintained our focus on the pre-registered primary outcome, following Cochrane Collaboration guidelines to meet the highest criteria for self-reported/subjective stress outcomes by searching trial registers for unpublished studies. There is limited search functionality on trial registers and time involved in contacting researchers for trial data. Moreover, as mentioned above, some previous reviews have not searched unpublished, grey literature before and there are less data available on breathwork and self-reported/subjective stress, in comparison to self-reported/subjective anxiety and depression. In brief, given our focus on stress (paired with time and resource constraints), we conducted the most robust search possible for the primary outcome whilst secondary outcomes only included published data—and we were explicit about this from pre-registration onwards.

### Inclusion and exclusion criteria

Inclusion criteria were that studies: (1) were published in the English language, (2) included a breathwork intervention where breathwork formed 50% or more of the intervention (and home practice/self-practice, if any), (3) were RCTs, (4) included an outcome measure of self-reported/subjective stress, anxiety, or depression, (5) included an adult participant sample 18 + years of age. For the five databases, studies with abstracts that did not include either the primary outcome keyword (stress), or a secondary outcome keyword (anxiety or depression), were excluded. For the two registers, if it was clear from the summary information that trials did not comprise the primary outcome of stress, they were excluded. As mentioned above, stress is a transdiagnostic health variable, relevant across various (clinical and nonclinical) populations and conditions, hence it was our primary interest. Additional rationale included the fact that there is far more limited research literature available on self-reported/subjective stress and breathwork (as opposed to anxiety and depression) and, since this was the primary outcome, because fewer (published) data were available, and to make the secondary search (which was only used in the present study to contextualise findings) more feasible, we used the referred search strategy, as this allowed us to find more information on stress from unpublished sources.

For all electronic repositories, studies with control conditions that comprised components of breathwork were excluded, except for studies which had time-points wherein data were collected before controls participated in breathwork (i.e., crossover RCTs). Only non-breathwork controls were used as post-intervention comparisons. Studies with interventions that comprised of equipment (oronasal or otherwise) which physically altered and/or assisted breathing activity were excluded. Breathwork was operationalised as techniques which involved conscious and volitional control or manipulation of one's breath (depth, pattern, speed or otherwise) through deliberate breathing practices. Interventions that affected breathing as a by-product, e.g., mindfulness, singing, and aerobic exercise, were excluded.

### Review strategy and study selection

The first author conducted the search and initial screening against eligibility criteria along with full-text screening. Records were then screened, excluding reports based on review of titles and keywords in abstracts or summary information (for trials), or if the inclusion criteria were not met. Remaining reports were sought for retrieval and the full-text reports assessed for eligibility, before final eligibility decisions were made. Further identification of studies comprised forward and backward citation searching via Google Scholar and reference lists, respectively, of the final reports included from the database/registry search. For inter-rater consistency purposes, one of the authors (JMM) checked a random sample (10% of reports) after duplicates had been removed. Furthermore, where GWF was unsure after full-text screening, they consulted authors KC and CS to come to a collective decision on eligibility. Any discrepancies between authors were resolved by discussion and reaching consensus.

### Data extraction

Our primary outcome was self-reported/subjective stress. Secondary outcomes were self-reported/subjective anxiety, depression, and global mental health (where two or more of stress, anxiety and depression were combined into a total measure without providing subscale data). We extracted the following data across the studies’ conditions: sample sizes, means, and standard deviations of outcome scores post-intervention (timepoint 1—T1, where T0 is pre-intervention/baseline) along with at latest follow-up where possible (a true follow-up was classed as when participants no longer received any instruction for the breathwork intervention). Where studies involved crossover designs, the midpoints were categorised as post-intervention (before the control group started the breathwork given initially to the intervention group). For studies which required multiple groups’ mean and standard deviation (M ± SD) scores to be combined, or for just SDs to be calculated, these were calculated in accordance with the Cochrane Collaboration handbook^48^. For example, calculating SDs from Ms and 95% confidence intervals (CIs) or combining multiple groups’ M ± SD scores if two or more groups completed an intervention that involved breathwork (but the study still comprised a non-breathwork control).

### Risk of bias and quality assessment

The most recent, revised Cochrane Collaboration’s tool for assessing risk of bias in randomised trials (RoB 2)^49^ was used for analysing studies on the primary outcome measure of self-reported/subjective stress. The studies were analysed across the following five domains for the stress outcomes: randomisation process, deviations from intended interventions, missing outcome data, measurement of the outcome, and selection of the reported result. Each domain produced an algorithmic judgement of “low risk of bias”, “some concerns”, or “high risk of bias”, resulting in an overall risk of bias judgement. For further inter-rater consistency purposes, both JMM and GWF completed bias scoring using RoB 2 on all included studies for stress, with any discrepancies resolved via discussion.

### Data synthesis and analysis

To evaluate whether breathwork can effectively lower stress compared to non-breathwork controls and to quantify the estimation we ran a quantitative synthesis meta-analysis using standardised mean differences and a random-effects model. This used aggregate participant data of M ± SD scores on stress outcome measures for intervention and control conditions of each study at post-intervention (T1), along with the groups’ sample sizes. We also conducted a sensitivity analysis by removing one study at a time, to evaluate the robustness of effects. Separate random-effects meta-analyses were run for the secondary outcomes. The software Review Manager (RevMan) version 5.4^50^ was used. For the between-group effect sizes (ESs) we computed Hedges’ *g*, based on the standardised between-group difference at post-intervention considering sampling variance among groups; an ES of 0.2 is classed as small, 0.5 medium and 0.8 large^51^. For each separate outcome, the ESs were calculated via comparison of post-breathwork intervention scores between the conditions. Intention-to-treat data were chosen over per-protocol data where available, since the former provides a more conservative estimate of between-group differences.

Heterogeneity of ESs variance was assessed using Cochran’s *Q*^52^ based on a chi-square distribution (*χ*^2^) and Higgins’ *I*^2^^53^*.* If *χ*^2^ is significant and an *I*^2^ index value is around 50%, this implies variance may be explained by variables other than breathwork and such statistical heterogeneity is moderate, respectively. A funnel plot was produced to examine publication bias for the primary outcome, and the software *R* (version 4)^54^ was used to explore asymmetry of the funnel plot via the Egger’s test^55^ (i.e., correlations between standard error and ESs). Moreover, Rosenthal’s fail-safe *N* was calculated (to estimate how many further studies yielding zero effect would be required to make the overall ES non-significant for stress)^56^. Kendall's *tau-b* (τ_B_) correlations were used to detect any potential relationships between ESs of breathwork on stress and: estimated total duration of intervention/home practice, total number of intervention/home practice sessions, and intervention/home practice session frequency. If intervention time was not provided by a study (where participants only had home practice), we used the minimum estimated home practice duration (recommended in the study) to gauge the approximate time taken for participants to ‘learn’ the breathwork technique. Minimum recommended duration was used for most conservative estimates, helping account for common attrition found across behavioural studies.

Lastly, subgroup analyses were run for stress, again using a random-effects model. These subsets included: health status of population (physical, nonclinical, or mental health), technique type (fast or slow-paced breathing) and delivery method of the breathwork intervention (individual, group, or a combination of both, and remote (self-help), in-person, or combination) along with the type of control group (active or inactive; in line with Cochrane Collaboration guidelines^48^), and outcome measure used (scale).

## Results

### Search results

**Figure 1:**
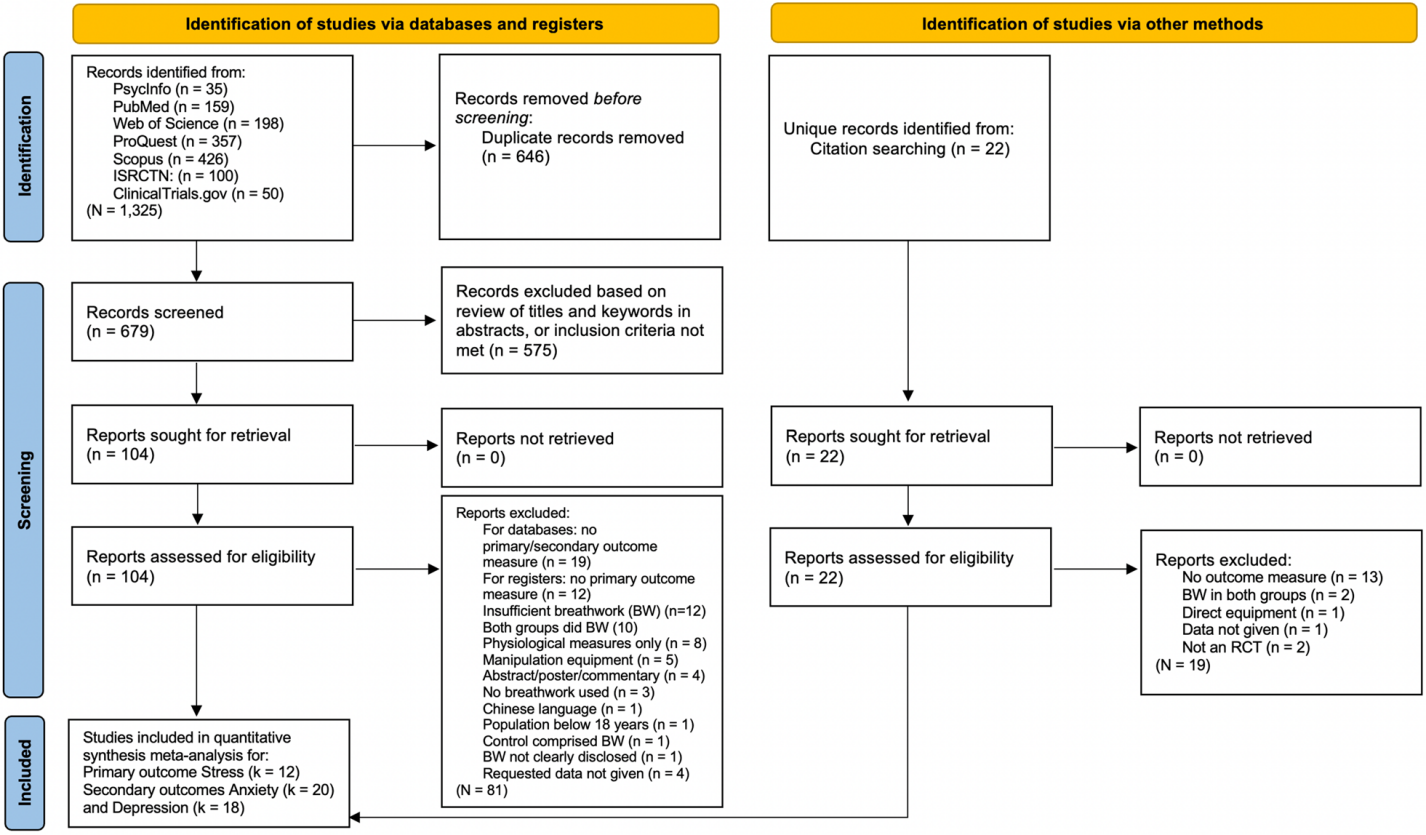
PRISMA flow diagram showing the identification of eligible studies via databases, registers, and citation searching. Self-reported/subjective stress was the primary outcome for the quantitative synthesis random-effects meta-analysis. Total number of included studies was 26. Trial registries searched primary outcome only.

As shown in Fig. 1, the search produced 1325 results: 1175 and 150 records from databases and registers, respectively. After duplicates were removed, the titles and abstracts (or summary information for registers) of 679 records were screened. During screening, the eligibility of 11% of reports were decided collectively among GWF, KC, and CS. All studies included by GWF were checked by KC and CS to ensure none were incorrectly included. One particular study^57^ that comprised a global mental health measure only had to be excluded as there were insufficient studies to reliably interpret results (*n* < 5)^58^—the only other available was Goldstein et al.^59^ (which also included a measure of self-reported/subjective stress). Accordingly, the global mental health secondary outcome was dropped from the analysis.

Further data were required for eight reports; corresponding authors were contacted, and data from four studies were retrieved, but not the remaining half^60–63^ subsequently excluded from the analysis. Thus, a total of 104 reports were screened and 81 were excluded, leaving 23. As a result of citation searching, a further three studies were included. Of the 26 total reports included in the quantitative synthesis meta-analyses, stress comprised 12 studies^59,64–74^. Secondary outcomes of self-reported/subjective anxiety and depression comprised of 20 studies^64–70,72–84^ and 18 studies^64–67,69–72,74,78–82,85–88^, respectively. Please see Online Appendix B for more information on the secondary outcomes.

### Summary of findings for stress

In terms of data extraction, all studies provided raw M ± SD scores apart from two^55,56^ where estimated marginal M ± SDs were given (raw data was requested from corresponding authors but could not be obtained). One study^65^ required SDs from Ms and 95% confidence intervals (CIs) provided, both of which were calculated in accordance with Cochrane Collaboration guidelines^48^. Furthermore, another study^70^ required two groups’ M ± SD scores (there was one control group and two intervention groups) to be combined and two further studies^64,71^ involved crossover designs (hence data were extracted at the midpoints of each study before controls started the breathwork intervention). Analyses of follow-up scores were not possible for self-reported/subjective stress as there were insufficient studies for results to be reliably interpreted^58^.

The 12 studies included in the meta-analysis for the primary outcome of stress were completed from 2012 to 2021 (seven, or 60%, were conducted from 2020 onwards). Half of these studies were conducted in the US^59,64–66,68,74^, two in India^71,72^, one globally^73^, and one each in: Israel^70^, Turkey^67^, and Canada^69^. The average age was 41.7 (± 8.47) and 75% identified as female, since the largest study^68^ was for women only. Attrition rates (after the breathwork intervention began) ranged from 3 to 40%. Participant sample sizes ranged from 10 to 150, with the total number of participants analysed being 785. The number of participants randomised to a breathwork intervention or control condition was 417 and 368, respectively. The minimum total estimated durations of an intervention/home practice ranged from 80 to 5625 min.

Half of the studies comprised physical health, five nonclinical, and one mental health samples. Ten and two studies comprised interventions with a primary focus on slow-paced breathing and fast-paced breathing, respectively. Seven were individual-based interventions, four taught to groups, and one a combination of both modes. Half were remote/self-help interventions, five in-person, and one combination. Seven and five studies had inactive and active control groups, respectively. Eight studies used the perceived stress scale (PSS)^89^, three used the stress subscale from the depression anxiety stress scale (DASS)^90^, and one used the perceived stress questionnaire (PSQ)^91^.

### Risk of bias for stress

**Figure 2:**
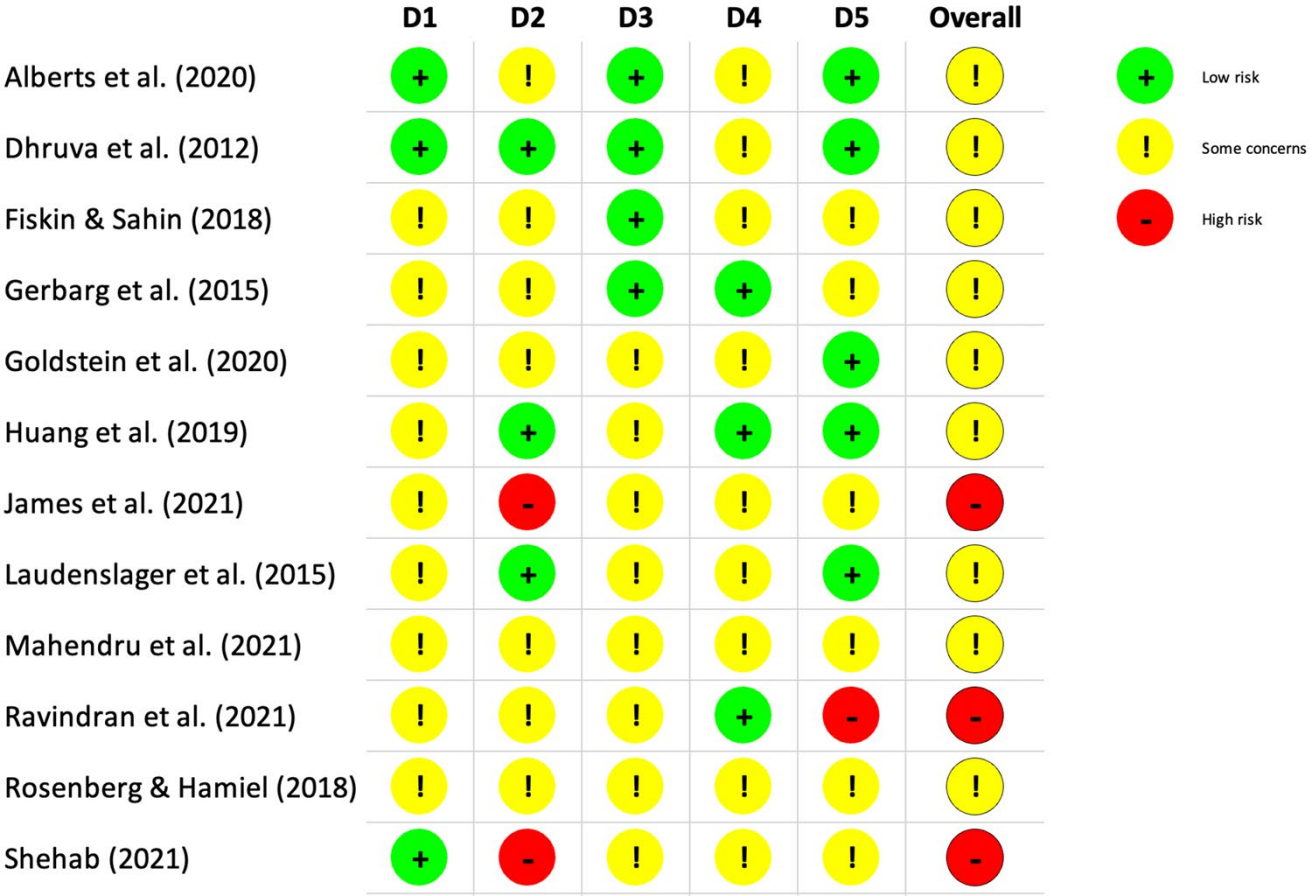
Risk of bias scoring using Cochrane Collaboration’s RoB 2 tool. Green and red colours correspond to low and high risk of bias, respectively. Yellow represents some concerns. *D1* Randomisation process, *D2* Deviations from the intended interventions, *D3* Missing outcome data, *D4* Measurement of the outcome, *D5* Selection of the reported result.

Risk of bias scoring for the 12 studies on the primary outcome is reported using RoB 2 in Fig. 2. Three studies’ overall assessment were algorithmically scored as being at high risk of bias, with domain two (deviations from the intended interventions) contributing to most bias. The remaining nine studies’ overall risk of bias were algorithmically scored as having some concerns. Only one study did not disclose how randomisation was conducted. Most of the domains, from randomisation to selection of the reported result, were scored as having some concerns or low risk of bias. We did not find reported adverse events or lasting bad effects directly attributed to breathwork interventions; four studies (six in total including secondary outcome studies) actively reported on this. Nonetheless, regarding safety and tolerability, a small subgroup of participants in Ravindran et al.’s study^71^ focusing on fast-paced breathwork in unipolar and bipolar depression reported side effects such as hot flushes, shortness of breath and/or sweating. However, these participants opted to continue the intervention and no participants dropped out of the breathwork group due to adverse effects.

### Breathwork and stress

**Figure 3:**
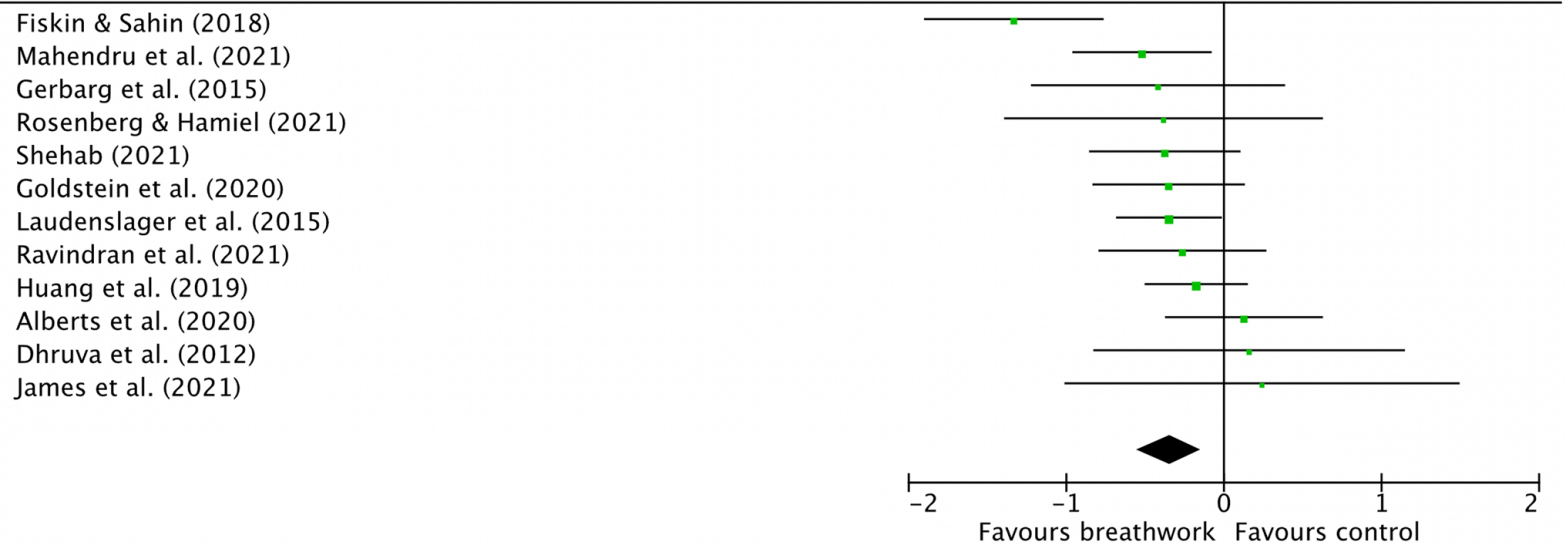
Forest plot comparing breathwork interventions to non-breathwork control groups on primary outcome of self-reported/subjective stress at post-intervention. Squares and their size represent individual studies and their weight, respectively. Lines through squares are 95% CIs and diamond is the overall effect size with 95% CIs. More negative values denote larger effect of breathwork on self-reported/subjective stress in comparison to control condition. Effect sizes calculated using Hedges’ *g*. Figure produced using RevMan v5.4.

As shown in Fig. 3, the random-effects meta-analysis *(k* = 12) displayed a small-medium but significant post-intervention between-group ES, *g* = − 0.35 [95% CI − 0.55, − 0.14], *z* = 3.32, *p* = 0.0009, denoting breathwork was associated with lower levels of self-reported/subjective stress at post-intervention than controls. There were insufficient studies including follow-up measures for a meta-analysis. Heterogeneity was moderate but non-significant, *χ*^2^_11_ = 19, *p* = 0.06, *I*^2^ = 42%. Via removing one individual study at a time, the ES of breathwork on stress ranged from − 0.27 to − 0.39 and remained significant in all cases. Initial visual inspection of the funnel plot in Online Appendix C suggested some skew due to studies with small samples; however, the Egger’s test was non-significant, *z* = 0.03, *p* = 0.947, indicating a low chance of publication bias. Fail-safe *N* analysis denoted that a further 69 studies yielding zero effect would need to be added to make the overall ES non-significant for stress. On removal of the one potential outlier^67^ the ES remained significant but became smaller: − 0.27. On removal of the two studies using estimated marginal M ± SDs, the ES remained significant and became larger: − 0.40.

### Subgroup analyses for stress

As displayed by Table 1, we conducted five sub-analyses for the primary outcome self-reported/subjective stress. There were no significant differential effects between subgroups.

**Table 1:** Subgroup analyses on effect of breathwork on self-reported/subjective stress at post-intervention. *p < 0.05, **p < 0.01*.* Heterogeneity not applicable where one study (*n* = 1) analysed. There were no significant differences between subgroups.

Subgroup	Type	*N*	Hedges’ *g*	95% *CI*	*Z*	Heterogeneity	
						*χ*^2^	*I*^2^
Population health	Physical	6	− 0.38	− 0.80, 0.04	1.79	18.1**	72%
	Nonclinical	5	− 0.34	− 0.56, − 0.11	2.94**	0.87	0%
	Mental health	1	− 0.26	− 0.79, 0.26	0.98		
Breathwork intervention	Slow	10	− 0.35	− 0.61, − 0.10	2.74**	18.95*	52%
	Fast	2	− 0.31	− 0.66, 0.04	1.73	0.06	0%
Delivery modes/styles	Individual	7	− 0.26	− 0.43, − 0.09	3.02**	5.17	0%
	Group	4	− 0.59	− 1.10, − 0.09	2.29*	9.31*	68%
	Combination	1	− 0.16	− 0.82, 1.14	0.75		
	Remote	6	− 0.23	− 0.43, − 0.03	2.28*	4.82	0%
	In-person	5	− 0.49	− 0.97, − 0.02	2.02*	11.3*	65%
	Combination	1	− 0.35	− 0.68, − 0.02	2.08*		
Control group	Active	5	− 0.26	− 0.48, − 0.04	2.28*	0.61	0%
	Inactive	7	− 0.37	− 0.73, − 0.02	2.07*	17.6**	66%
Stress outcome	PSS	8	− 0.22	− 0.39, − 0.06	2.65**	4.38	0%
	DASS	3	− 0.79	− 1.40, − 0.18	2.54*	5.68	65%
	PSQ	1	− 0.42	− 1.22, 0.38	1.03		

There was a significant effect of breathwork on stress in nonclinical samples, but not in mental (only one study) or physical health populations. Moreover, significant effects were yielded when breathwork was primarily focused on slow-paced breathing (but not for fast-paced breathing), taught to individuals alone, and when taught to groups (but not in combination, which comprised only one study). There were also significant effects of breathwork on stress when the intervention was taught remotely, in-person, and using a combination of these two delivery methods. Significant effects existed for both active and inactive control groups. There were significant effects for studies which used PSS and DASS measures (but not the PSQ, used by only one study).

Heterogeneity was high for studies with physical health samples, slow-paced breathwork, when breathwork was taught to groups and in-person, plus those studies with inactive controls, and when stress was measured by using the DASS, suggesting potential moderating factors that were not accounted for by the subgroup analyses. There was no significant correlation between estimated total duration of breathwork intervention/home practice and ES (*n* = 12) *τ*_*B*_ = − 0.05, *p* = 0.418, number of intervention/home practice sessions and ES for stress (*n* = 12) *τ*_*B*_ = − 0.28, *p* = 0.107, nor for intervention/home practice session frequency and ES (*n* = 12) τ_B_ = − 0.17, *p* = 0.224.

### Breathwork and secondary outcomes

In terms of data extraction, one study^79^ had a measure with positively scored anxiety and depression subscales; accordingly, we subtracted the subscale score from the maximum score to reverse the polarity of the measure without changing the magnitude of difference. Another study^88^ required two groups’ M ± SD scores to be combined. Analysis of follow-up scores were not possible for secondary outcomes as there were insufficient studies^58^ (*n* < 5). Forest plots for the secondary outcomes are reported in Online Appendix D. Random-effects analysis for anxiety (*k* = 20) showed a significant small-medium between-group ES in favour of breathwork, *g* = − 0.32 [95% CI − 0.48, − 0.16], *z* = 3.90, *p* < 0.0001, with moderate and significant heterogeneity, *χ*^2^_19_ = 38.62, *p* = 0.005, *I*^2^ = 51%. Sensitivity analysis showed ESs ranging from − 0.29 to − 0.34, significant in all cases. No individual study was responsible for the significant heterogeneity. Random-effects analysis for depression (*k* = 18) displayed a significant small-medium ES in favour of breathwork, *g* = − 0.40 [95% CI − 0.58, − 0.22], *z* = 4.27, *p* < 0.0001, and heterogeneity was moderate and significant, *χ*^2^_17_ = 40.5, *p* = 0.001, *I*^2^ = 58%. Sensitivity analysis showed ESs ranging from − 0.35 to − 0.44, significant in all cases. On removal of two potential outliers^85,88^, the ES remained the same. No single study was responsible for the significant heterogeneity.

## Discussion

### Breathwork and stress

We conducted the first comprehensive systematic review and meta-analysis of RCTs on the effect of breathwork on self-reported/subjective stress, analysing 12 studies which comprised a total of 785 participants. Breathwork yielded a significant post-intervention between-group effect of breathwork on stress compared to non-breathwork controls, denoting breathwork was associated with lower levels of stress than controls.

Statistical heterogeneity was moderate but not significant, meaning variance in ESs was likely explained by breathwork rather than other variables, although this non-significance could also be a consequence of the low number of studies included. This small-medium ES should be interpreted in the light of moderate risk of bias overall for the 12 studies. More than half of the studies included in our meta-analysis for stress were completed from 2020 onwards, suggesting a recent emergence of research into breathwork, which may have been accelerated by the covid-19 pandemic. Research on breathwork could be likened to that of meditation, which received an unprecedented surge in scientific exploration two decades ago^92^. We may be at a similar cusp with breathwork and anticipate considerable growth in the field. Given the close ties of breathwork to psychedelic research^93^, which is growing rapidly, this could accelerate growth further.

Regarding subgroup analyses for self-reported/subjective stress, heterogeneity was significant for studies with physical health samples, slow-paced breathwork interventions, inactive control groups, along with studies when breathwork was group-based and in-person. At present, there are too few studies within the sub-analyses to address this issue of statistical heterogeneity. Overall, point estimates were similar and sample sizes were small, hence where results were non-significant, it is unclear whether there was genuinely no effect, or lack of statistical power. Furthermore, no significant differential effects across subgroups were observed, but this could also be the result of the scarce number of studies.

While nonclinical samples showed a significant effect on self-reported/subjective stress outcomes and physical and mental health samples did not, between-subgroup differences were non-significant and the point estimates for these subgroups were similar (ranging from ES = 0.26–0.38). These findings could mean that breathwork is not effective for physical/mental health populations, however, it is also possible that this analysis was underpowered to detect effects given the relatively small number of studies contributing to the subgroups, as we have already mentioned. There were only two studies primarily focused on fast-paced breathwork and stress, insufficient to make a meaningful comparison with the ten studies primarily focused on slow-paced breathwork. Interestingly, delivery modes and styles did not seem to influence the results, which may suggest breathwork can be learned through several different formats. Half of the studies’ interventions were delivered remotely without instructors (self-help), hence breathwork could potentially be widely disseminated and thus accessible and probably scalable. The results were significant for both active and inactive controls, although it would be expected that breathwork would have less effect compared to active controls. This could be due to poor quality of the active controls. Lastly, results were significant for two of three stress outcome measures, most likely due to them being psychometrically well-validated—only one study used the third measure (PSQ).

Concerning dose–response, although associations were in the expected direction, there were no significant correlations between the minimum estimated durations of breathwork intervention/home practice and ES, for all outcomes. This apparent absence of dose–response effects was surprising as increased practice time might be expected to be associated with greater benefit, however compliance to intervention home practice was not reported for many studies and so true dose–response analysis was not possible. Moreover, intention-to-treat analysis data were used for the most conservative estimates of effect. Dhruva et al.’s study^64^ included in our meta-analysis specifically investigated dose–response effects, finding a positive relationship between total amount of breathwork intervention/home practice and improvement in quality of life and chemotherapy-associated symptomology—there was a significant decrease in anxiety for each hour increase in breathwork. Alternatively, this could be indicative of breathwork being possibly able to help quickly, as suggested in very recent literature whereby just one session of slow, deep breathing had beneficial effects on anxiety and vagal tone in adults^94^, with vagal tone being measured, albeit indirectly, through HRV^6^. This may be likened to ‘micro dosing’ breathwork, similar to single session mindfulness meditation practices^95^.

The meta-analysis results are largely consistent with and extend upon previous work. For instance, our findings are somewhat in line with Malviya et al.’s recent review which provides some support for breathwork’s effectiveness in alleviating stress^43^. However, this review only included two studies for stress, one of which comprised of both groups incorporating breathing practices (and was thus excluded from our meta-analysis). Hopper et al.’s systematic review on diaphragmatic breathing found just one RCT for stress, however this used physiological measures^42^. Nonetheless, this study showed that the stress hormone cortisol was lower in people undergoing slow-paced breathwork compared to controls^96^. In a different study^38^, participants administered with bacterial endotoxin (*E. coli*) who performed fast-paced breathwork had higher spikes of cortisol compared to non-breathwork controls, during the intervention, but a quicker recovery and stabilisation of cortisol levels after cessation of breathwork. This could be another mechanism of action warranting further investigation.

### Breathwork, anxiety and depression

Furthermore, meta-analyses comprising 20 and18 studies run for secondary outcome measures of self-reported/subjective anxiety and depressive symptoms, showed that breathwork interventions also yielded significant small-medium ESs in comparison to controls, favouring breathwork (see Online Appendix D for results). However, heterogeneity was significant for both outcomes, meaning the variance in ESs may be due to other variables apart from breathwork. Thus, these ESs should be interpreted with caution and need further research. As per Malviya et al.’s review^43^, greater support was offered for breathwork in alleviating anxiety and depressive symptoms (eight studies for both outcomes). The review deemed findings pertaining to the efficacy of breathwork in decreasing anxiety and depression as promising. This was also consistent with Zaccaro et al.’s review findings on slow breathing (15 studies—no RCTs), that had lower self-reported anxiety and depression, possibly linked to increased HRV measured during interventions^4^. Ubolnuar et al.’s review of breathing exercises for COPD found no significant effect on anxiety and depression from a subgroup meta-analysis of two RCTs, however the interventions used for both were singing classes^39^. Nonetheless, a recent meta-analysis by Leyro et al. of 40 RCTs on interventions for anxiety, which comprised a respiratory component (ranging from diaphragmatic breathing to capnometry assisted respiratory training), showed such treatments were associated with significantly lower symptoms of anxiety compared to control groups^41^. Though non-respiratory controls were used, respiratory components did not have to form a significant part of the intervention, thus it is less possible to tease out the effects of such techniques. While some interventions used physically altering equipment such as training of musculature involved in respiration, this might provide further potential for breathwork-related work in clinical conditions.

### Comparison to stress-reduction interventions

Through estimating statistically significant differences and 95% CIs among studies^97^, in comparison to interventions for stress, our findings suggest that breathwork might be associated with similar—and non-significantly different—effects. For instance, Heber et al.’s meta-analysis on computer- and online-based stress interventions, including CBT and third-wave CBT (e.g., inclusion of meditation, mindfulness, or acceptance of emotions) compared to controls in adults, found moderate effects on stress, *d* = 0.43 [95% CI 0.31, 0.54], anxiety, *d* = 0.32 [95% CI 0.17, 0.47], and depression, *d* = 0.34 [95% CI 0.21, 0.48]^98^. Each of these effects overlap more than 25% with the width of either interval in our results for breathwork, denoting no indication of a clinically relevant difference between the interventions. Similar meta-analytic findings concerning effects on stress, anxiety and depression have been found for related and more analogous techniques such as mindfulness-based cognitive therapy and stress reduction (MBCT/MBSR)^99^ along with self-help (MBSH)^100^. While Pizzoli et al.’s recent post-intervention HRVB meta-analysis (14 published RCTs)^13^ found a significant effect on depression, another meta-analysis did not find a significant effect on stress, with the smallest ES being yielded for self-reported stress out of myriad outcomes^14^. Lastly, a meta-analysis of eight meta-analytic outcomes of RCTs on physical activity^99^ showed similar significant effects on depression and anxiety. While we are not proposing breathwork as a substitute for other treatments, it could complement other therapeutic interventions, potentially leading to additive effects of such health behaviours.

People with stress and anxiety disorders tend to chronically breathe faster and more erratically, yet with increased meditation practice, respiration rate can become gradually slower, potentially translating into better health and mood, along with less autonomic activity^92^. Positive impacts on HRV may partially explain some of the mechanisms behind mindfulness meditation^101,102^. However, the above approaches like MBCT/MBSR and HRVB may be less accessible. MBCT/MBSR teacher training takes at least one year while HRVB is routinely taught by a qualified healthcare professional; this is usually a prerequisite and most certified biofeedback therapists are habitually licensed medical providers, including general practitioners, psychiatrists, dentists, nurses, and psychologists^103^. MBCT/MBSR and HRVB therapist training includes theoretical/practical curricula, while breathwork teacher training can be more quickly and easily taught (i.e., over days and weeks) online and remotely to both healthcare professionals and the general population, thus potentially proving cost-effective.

Two of our studies used the only Food and Drug Administration-approved portable electronic biofeedback device, which encourages deep, slow breathing^103^. However, HRV can be improved in the same way (tenfold) by simply breathing at a rate around 5–6 breaths/min^104^ and some Zen Buddhist monks have been found to naturally respire around this rate during deep meditation^105^. It may be possible that breathing rate forms a key component of meditation’s known positive effects. Indeed, it has been shown that HRV can be modulated during the practice of meditation^106^. However, a recent meta-analysis on this exact matter found insufficient evidence suggesting mindfulness/meditation led to improvements in vagally mediated HRV, and more well-designed RCTs without high risk of bias are needed to clarify any such contemplative practices’ impact on this physiological metric^107^, along with potential mechanisms related to cortisol.

Traditional mindfulness-based programmes frequently involve meditation requiring observation of the breath, using it as an object of awareness, not voluntary regulation of respiration like in breathwork. Such breath-focus may be a key active ingredient and potential mechanism of action of the former contemplative practices, since highly experienced meditators have been found to breathe at over 1.5 times slower than nonmeditators, during meditation and at rest^108^. This translates into approximately 2000 less daily breaths for the former group of adept meditation practitioners (i.e., around 700,000 less breaths in a year), placing less demand on the ANS^92^. Meditation could also be complementary; voluntary upregulation of HRV through biofeedback may be improved by mental contemplative training^109^. While there is a possibility that it could simply be the cognitive-attentional components of both meditation and breathing practices that explain their effects, observation of the breath (i.e., most practices within mindfulness curricula) versus control of the breath (i.e., breathwork) warrants nuanced investigation.

### Strengths, limitations and future directions

Our systematic review searched published, unpublished and grey literature across numerous electronic databases and the meta-analysis comprised several very recent RCTs with well-validated measures of self-reported/subjective stress. However, like most systematic reviews in this field, given the small sample size (likely due to the recent phenomena of breathwork in the West) and moderate risk of bias across the studies included in our meta-analysis, our results should be interpreted cautiously. Future studies exploring breathwork’s effectiveness should aim for research designs with low risk of bias. While this review attempted to bridge the gap and unify both old and new research, future low risk-of-bias studies are now needed in order to draw definitive conclusions of breathwork’s impact on mental health. There were also not enough studies for valuable subgroup comparisons, and therefore we did not identify any potential sources of heterogeneity. Furthermore, secondary outcomes were not scrutinised with the same level of detail as the primary outcome, as they were only used to provide complementary context and a bigger picture around stress and mental health in general.

Our meta-analysis is the first review of breathwork’s impact on self-reported/subjective stress and its therapeutic potential, and combining this quantitative synthesis of psychological effects of breathwork with other syntheses, i.e., of physiological effects^4^, could help build a stronger psychophysiological model of breathwork’s efficacy along with more robust mechanisms of action. Studies could use stress subscales in DASS as standard in addition to the anxiety/depression scales, as this could be important for nonclinical and subclinical populations experiencing stress and allow for direct comparison of effects across clinical/nonclinical populations. Additionally, psychophysiological RCTs combining both subjective and objective measures in line with proposed mechanisms of action (i.e., self-reported stress and ECG HRV/respiration rate measurements) should be conducted, along with further imaging (MRI, EEG, NIRS, etc.) studies on various breathwork techniques (only one fMRI study was available in Zaccaro et al.’s review^4^). This could help better determine modalities and underlying principles of different breathwork techniques. Though validated scales were used for stress in the meta-analysis, our review lacks objective outcomes, which increases risk of bias further.

Comparison groups promoting observation versus control of the breath could yield interesting findings when exploring any differences between the effects of meditation and breathwork. However, robust scientific methods that align well with current methodological demands on meditation and contemplative psychological science^110^ should be implemented. There was also limited scope to report on follow-up effects, hence more studies could include true follow-up timepoints and longitudinal designs, now more common in meditation and contemplative science research. On top of this, there could be cross-cultural differences in response to breathwork (i.e., between Eastern and Western modalities) which could be explored by future research, along with searching non-English language literature. There could also be differences between age categories (including children); this meta-analysis focused solely on adults across a broad age-range. Lastly, more studies should report on adverse events and lasting bad effects, with further research needed to gauge the safety profile of fast-paced breathwork in particular, so it not administered blindly to potentially vulnerable populations.

### Clinical implications

For stress, though not many studies monitored home practice/self-practice, engagement with interventions appeared good, none reporting adverse effects directly attributed to breathwork. This suggests breathwork has a high safety profile and slow-paced breathing techniques can be recommended to subclinical populations or those experiencing high stress. However, regarding clinical populations, the findings from our meta-analysis show non-significant effects for mental and physical health populations, hence it could be premature to recommend breathwork in these contexts. If breathwork can indeed provide therapeutic benefit to specific populations, conducting research with strong, low risk-of-bias design is essential to understanding if breathwork is genuinely effective or not. Ethicality should always take centre stage, with first doing no harm being the priority. Nonetheless, in nonclinical settings (excluding those predisposed to mental and physical health conditions), the low cost and risk profiles make breathwork (primarily focused on slow-paced breathing), scalable, with evidence from this meta-analysis that some techniques can potentially be self-learned, not requiring an instructor in real-time. Providing future robust research shows positive effects of breathwork, only then can an evidence-based canon be borne out of breathwork, using standardised and manualised materials for both training and practicing various secular, accessible techniques. However, there is a possibility rigorous research demonstrates that breathwork is not effective. Moreover, precaution must be exercised at all times; clinicians should consider for the individual whether breathwork may exacerbate the symptoms of certain mental and/or physical health conditions (cf. Muskin et al.^111^).

## Conclusions

More accessible therapeutic approaches are needed to reduce, or build resilience to, stress worldwide. While breathwork has become increasingly popular owing to its possible therapeutic potential, there also remains potential for a miscalibration, or mismatch, between hype and evidence. This meta-analysis found significant small-medium effects of breathwork on self-reported/subjective stress, anxiety and depression compared to non-breathwork control conditions. Breathwork could be part of the solution to meeting the need for more accessible approaches, but more research studies with low risk-of-bias designs are now needed to ensure such recommendations are grounded in research evidence. Robust research will enable a better understanding of breathwork’s therapeutic potential, if any. The scientific research community can build on the preliminary evidence provided here and thus, potentially pave the way for effective integration of breathwork into public health.

## Supplementary Information

Supplementary Information.

## Data Availability

The datasets used and/or analysed during the current study available from the corresponding author on reasonable request.
