# Supplementary material for: Effect of breathwork on stress and mental health: A meta-analysis of randomised-controlled trials
Source: Sci Rep. 2023 Jan 9;13:432. doi: 10.1038/s41598-022-27247-y (PMC9828383; doi:10.1038/s41598-022-27247-y)
Supplement: Supplementary file 1 — Supplementary Information. [file 41598_2022_27247_MOESM1_ESM.pdf]

Effect of breathwork on stress and mental health: A meta-analysis of randomised-controlled trials

Mr Guy William Fincham[1*], MSc, Prof Clara Strauss[1,2], DClinPsy, PhD, Dr Jesus Montero-Marin[3,4,5*], DClinPsy, PhD, Prof Kate Cavanagh[1,2], DClinPsy, PhD

[1] Department of Psychology, University of Sussex, Brighton, UK
[2] Research & Development Department, Sussex Partnership NHS Foundation Trust, Brighton, UK
[3] Department of Psychiatry, Warneford Hospital, University of Oxford, Oxford, UK
[4] Teaching, Research & Innovation Unit, Parc Sanitari Sant Joan de Déu, Barcelona, Spain
[5] Consortium for Biomedical Research in Epidemiology & Public Health (CIBER Epidemiology & Public Health—CIBERESP), Madrid, Spain
*Corresponding authors

Appendices A, B, C, D

Appendix A.

Table A. Search strategy for published and unpublished scientific literature across five databases (primary outcome of self-reported/subjective stress and secondary outcomes) and two trial registries (primary outcome only). Wildcard search term (*) could not be used in trial registers.

**Database**	**Search**	**Filters applied**
PsycInfo	*Title*: ("Breath*" OR "Respir*") AND ("Random*" OR "RCT"). *Anywhere/all fields*: ("Stress*" OR "Anxi*" OR "Depress*")	None
PubMed	*Title*: ("Breath*" OR "Respir*") AND ("Random*" OR "RCT"). *Anywhere/all fields*: ("Stress*" OR "Anxi*" OR "Depress*")	None
Web of science	*Title*: ("Breath*" OR "Respir*") AND ("Random*" OR "RCT"). *Anywhere/all fields*: ("Stress*" OR "Anxi*" OR "Depress*")	None
ProQuest	*Title*: ("Breath*" OR "Respir*") AND ("Random*" OR "RCT"). *Anywhere/all fields*: ("Stress*" OR "Anxi*" OR "Depress*")	None
Scopus	*Title*: ("Breath*" OR "Respir*") AND ("Random*" OR "RCT"). *Anywhere/all fields*: ("Stress*" OR "Anxi*" OR "Depress*")	None
**Trial registry**		
ISRCTN	*Anywhere*: ("Breath" OR "Breathwork" OR "Breathing" OR "Breathe" OR "Respiration" OR "Respire" OR "Respiring" OR "Respiratory") AND "Stress"	Completed With results
ClinicalTrials.gov	*Intervention/treatment*: "Breath" OR "Breathwork" OR "Breathing" OR "Breathe" OR "Respiration" OR "Respire" OR "Respiring" OR "Respiratory". *Outcome measure*: "Stress"	Completed With results

Appendix B.

Table B. Summary information of all the included RCTs in the meta-analyses for all outcomes. * = Study included primary outcome of self-reported/subjective stress. The 26 studies were completed from 2000 to 2021 and spanned 11 countries, half of which were based in the US and India. Study duration ranged from one day to seven months. The average age was 44, with 1,783 participants in total included in the meta-analyses, over half of whom identified as female. Roughly half of the participants were randomly allocated to a breathwork intervention (n=910) or non-breathwork control (n=873). Attrition rates (after the breathwork intervention began) ranged from 0-45%. In line with the PICO framework: 14 studies contained physical health populations, eight non-clinical and four mental health. While some interventions comprised various techniques, 20 were classed as having a primary focus on slow-paced breathwork, and six were classed as having a primary focus on fast-paced breathwork. Moreover, 12 studies were group-based, 12 individual-based, and two a combination of both; 15 were in-person, nine remotely or online, and two a combination of these modes. The minimum total estimated durations of an intervention/home practice ranged from 1-6,810 minutes; the minimum total estimated number of intervention/home practice sessions ranged from 1-173. Although intervention/home practice sessions differed in length, the intervention/home practice session frequency ranged from 2-21.5 times per week. Sixteen studies had inactive control groups and 10 had active controls. All studies for stress contained psychometrically well-validated outcome measures.

**Authors**	**Country**	**Population**	**Age M (SD)**	**Sample N (I/C)**	**Gender (F/M)**	**Intervention**	**Control**	**Measure used**	**Intervention duration & time-points of measures**
Alberts et al. (2020)*	Canada	Childhood cancer survivors	44.1 (8.7)	32/33 (IA: 31/32)	35/30	Spire Stone; asked to practice one of the app's brief deep-breathing exercises and/or take three deep breaths if app sent notification	Wait-list	PSS-10, GAD-7, PHQ-8	T0; pre-I, T1; post-I 31 days after T0
James et al. (2021)*	Global	Alzheimer's disease caregivers	60.3 (7.4)	14/14 (IA: 5/5)	14/5	Heart Lock-In heart-focused breathing technique & meditation - slow deep breathing shifting focus to heart area and inducing positive thoughts/emotions	Wait-list	PSS-10, PROMIS	T0; pre-I, T1; post-I 2 weeks after T0
Mahendru et al. (2021)*	India	Asymptomatic/mildly symptomatic SARS-CoV-2 infected patients isolating	35.5 (9.98)	42/42 (IA: 42/42)	27/57	Anulom vilom / Nadi shodhan pranayama [alternate nostril breathing] & meditation	Standard care	DASS-21	T0; pre-I, T1; post-I 1 week after T0
Ravindran et al. (2021)*	India	Outpatients with unipolar or bipolar depression	39.7 (11.9)	53/19 (IA: 53/19)	57/15	Breath control (pranayamas) - Ujjayi, Cyclical rapid breathing Kapalabhati - 40-60 breaths/minute, 60-80 breaths/minute, 80-100 breaths/minute	Psychoeducation protocol for unipolar and bipolar depression	PSS-10, MADRS	T0; pre-I, T1; post-I 8 weeks after T0
Rosenberg & Hamiel (2021)*	Israel	University students during exam period	25.1 (2.6)	13/13/13 (two I's) (IA: 9/8/5)	21/13	I-1: CalmiGo - slowly extending exhale length; I-2: Self-directed slow, deep belly (diaphragmatic) breathing	Psychoeducation handout focused on test anxiety	DASS-21, TAI	T0; pre-I, T1; post-I 2 weeks after T0

Fiskin & Sahin (2018)*	Turkey	Pregnant women with gestational diabetes	31 (3.2)	32/31 (IA: 30/30)	63 F	Diaphragmatic breathing exercises - inhaling through the nose and exhaling through the mouth	Standard care	DASS-42	T0; pre-I, T1; post-I 30 days after T0
Dhruva et al. (2012)*	US	Chemotherapy cancer patients	54.2 (13.25)	12/11 (IA: 8/8)	14/2	Pranayama yoga breathing (4 techniques - breath observation, ujjayi breathing [ocean breath / resistance breathing], kapalabhati [skull shining breath] - but in slow manner, nadi shodhana [alternate nostril breathing])	Usual care	PSS-10, HADS	n/a: I-group received pranayama during 2 consecutive cycles of chemotherapy; C-control group received usual care during first cycle & received pranayama during their second cycle of chemotherapy
Gerbarg et al. (2015)*	US	Inflammatory bowel disease patients	53.9 (15.2)	16/13 (IA: 14/11)	17/12	Breath-body-mind [various breath practices], movement, meditation, & coherent breathing [core technique] - 5 breaths/minute	Educational seminar on IBD	PSQ-30, BAI, BDI	T0; pre-I, T1; 6 weeks after, T2; post-I 24 weeks after T0 (in study: T1 and T2 classed as post-I & follow-up, respectively)
Goldstein et al. (2020)*	US	University students	20.7 (3)	55/53 (IA: 37/32)	50/19	Sudarshan Kriya Yoga (3/4 different rhythms of kapalabhati - slow, medium, medium-fast, fast) - by Art of Living Foundation	Wisdom on Wellness (WoW) psychoeducation stress management workshop	PSS-10	T0; pre-I, T1; 6 weeks after workshop, T2; post-I 3 months after T1 (in study, T1 and T2 classed as post-I & follow-up, respectively)
Huang et al. (2019)*	US	Female overactive bladder syndrome patients	61 (11.2)	79/82 (IA: 74/76)	161 F	RESPeRATE - synchronises breathing to tones with target of slow breathing, below 10 breaths/minute	RESPeRATE with music but without slow-paced breathing guidance	PSS-10, HADS	T0; pre-I, T1; post-I 12 weeks after T0
Laudenslager et al. (2015)*	US	Caregivers of patients undergoing bone marrow transplant	53.5 (12.3)	74/74 (IA: 73/71)	112/35	Psychoeducation paced respiration & relaxation (PEPRR) [using RESPeRATE] - less than 10 breaths/minute	Treatment-as-usual psychosocial care	PSS-10, STAI-S, CES-D	T0; pre-I prior to transplant), T1; one month after transplant, T2; post-I three months after transplant
Shehab (2021)*	US	Young adults with elevated stress	21.1 (2.86)	40/40 (IA: 33/37)	53/27	Resonant frequency [coherent] breathing training - 4.5-6 breaths/minute	Wait-list	PSS-10, STAI-S, BDI	T0; pre-I, T1; post-I 4 weeks after T0
Carter et al. (2013)	Australia	Vietnam war male veterans with PTSD	58.5 (4.2)	16/15 (IA: 14/11)	31 M	Sudarshan Kriya Yoga (adapted for veterans) [cyclical breathing - 3 different speeds]	Wait-list	CES-D	T0; pre-I, T1; 6 weeks after workshop, T2; post-I 6 months after T1 (in study, T1 and T2 classed as post-I & follow-up, respectively)
Evaristo et al. (2020)	Brazil	Outpatients with asthma	50.2 (9.5)	25/29 (IA: 25/29)	39/15	Breathing exercise program based on Pranayama Yoga breathing technique developed by 2 senior yoga instructors - breathing exercises aimed to stimulate nasal and diaphragmatic breathing, to increase expiratory time, to slow respiratory flow, and to regulate breathing rhythm	Aerobic training program - performed with an indoor treadmill initiated at 60% of heart rate (HR) recovery	HADS	T0; pre-I, T1; post-I 12 weeks after T0, T2; follow-up 3 months after T1
Novaes et al. (2020)	Brazil	Healthy young adults	25.1 (4.3)	15/15 (IA: 15/15)	15/15	Bhastrika pranayama - cycles of Kapalabhati, alternate nostril breathing and apnoea (breath holds)	Ludic cognitive activities such as crosswords, puzzles, domino, checkers, and card	STAI-S	T0; pre-I, T1; post-I 4 weeks after T0

							games, also in the presence of an instructor		
Pham et al. (2016)	Global	General population adults recruited online	n/a (18+)	31/32 (IA: 31/32)	31/31/1 other	Flowy app - mHealth game that engages users in a series of minigames using breathing retraining exercises / diaphragmatic breathing	Weekly newsletter with curated content on breathing retraining exercises, ''Flowy'' game development, mindfulness meditation, and similar content relevant to ''Flowy.''	GAD-7	T0; pre-I, T1; post-I 4 weeks after T0
Janakiramaiah et al. (2000)	India	Hospitalised untreated melancholic depressives	38.7 (7.4)	15/15/15 (two C's) (IA: 15/15/15)	45 M	Sudarshan Kriya Yoga with supine rest	C1: Electroconvulsive therapy (ECT), C2: imipramine (IMN)	HDRS	T0; pre-I after hospitalisation, T1; one week after T0, T2; two weeks after T0, T3; three weeks after T0, T4; post-I 4 weeks after T0
Kamath et al. (2017)	India	Healthy and yoga-naïve medical students	n/a (19-24)	15/15 (IA: 15/15)	21/9	Alternate Nostril Breathing before simulated public speaking test	Sitting in room before simulated public speaking test	VAMS-A	T0; 15 minutes pre-I, T1; post-I 55 minutes after T0 (single session)
Sureka et al. (2014)	India	Male prisoners with non-psychotic psychiatric disorders	36.1 (11.4)	116/116 (IA: 115/115)	230 M	Sudarshan Kriya and related practices (SK&P): 1) three staged Ujjayi breathing, (2) bellows breath (Bhastrika), (3) om chant, (4) SK and (5) ANB	Sit in armchair and pay gentle attention to breath	PGWB-Anx, PGWB-Dep	T0; pre-I, T1; post-I 6 weeks after T0
Valenza et al. (2013)	Spain	Male patients hospitalised with acute COPD exacerbation	75.2 (6.1)	23/23 (IA: 23/23)	46 M	Controlled breathing program included relaxation exercises, pursed lips breathing, and active expiration	Standard care	HADS	T0; pre-I after hospitalisation, T1; post-I at discharge
Chung et al. (2010)	Taiwan	Patients with coronary heart disease	71.5 (11.9)	33/39 (IA: 28/34)	19/43	Home-based deep-breathing programme: slow and deep diaphragmatic breathing technique at ~4-7 breaths/minute	Individual stress-management session and then weekly telephone support delivered by the same nurse who taught the breathing session	BDI	T0; pre-I, T1; 2 during treatment, T2; post-I 4 weeks after T0
Teng et al. (2018)	Taiwan	Heart failure patients	52 (13)	45/45 (IA: 41/43)	18/72	Walking with controlled breathing: abdominal deep breathing patterns were designed and taught; inhalation and exhalation times were targeted at a 1:2 ratio, and breathing rates were allowed to decrease to <10 times per min before walking would begin	Usual care	HADS	T0; pre-I, T1; post-I 12 weeks after T0
Tsai et al. (2015)	Taiwan	Chronic kidney disease patients on maintenance haemodialysis	n/a (18+)	32/32 (IA: 32/25)	n/a	Audio guided breathing training in a quiet room at the dialysis centre: Slow and deep nasal / diaphragmatic breathing technique at ~4-7 breaths/minute	Wait-list	BDI	T0; pre-I, T1; post-I 6 weeks after T0

Atilgan & Tuncer (2021)	Turkey	Mothers of children with special health care needs	34.9 (6.48)	23/23 (IA: 23/20)	46 F	Diaphragmatic and pursed lip breathing exercises with core stabilisation exercises (BSET)	Stabilisation exercises (SET)	STAI-S	T0; pre-I, T1; post-I 8 weeks after T0
Thomas et al. (2009)	UK	Asthmatics with impaired health status managed in primary care	46 (range: 33-57.3)	94/89 (IA: 94/89)	112/71	Exercises: diaphragmatic and nasal breathing techniques; Education: normal breathing and possible effects of abnormal "dysfunctional breathing" (over-breathing, mouth and upper chest breathing)	Asthma education: without providing personalised asthma advice	HADS	T0; pre-I, T1; one month post-I, T2; follow-up 6 months post-I
Van Denburg (2020)	US	Women undergoing MRI-guided breast biopsy	47.7 (12.1)	23/25 (IA: 23/25)	48 F	Breathing at a rate of 6 breaths/minute (breathing in for four counts and out for six counts [cadence breathing]) and music	Usual care (including music) during MRI-guided biopsy	STAI-S	T0; pre-I before biopsy; T1; post-I and biopsy (T0 and T1 completed in single session), T2; 24 hours post-I and biopsy

I: breathwork intervention group; C: non-breathwork control group; DASS: Depression Anxiety Stress Scales; PSS: Perceived Stress Scale; HADS: Hospital Anxiety and Depression Scale; PHQ: Patient Health Questionnaire depression scale; MADRS: Montgomery-Asberg Depression Rating Scale; TAI: Test Anxiety Inventory; BDI: Beck Depression Inventory; BAI: Beck Anxiety Inventory; VAMS-A: Visual Analogue Mood Scale-Anxiety; PGWB-Anx: The Psychological General Well-Being Index-Anxiety subscale; PGWB-Dep: The Psychological General Well-Being Index-Depressed Mood; GAD-7: Generalised Anxiety Disorder Assessment; HDRS: Hamilton Depression Rating Scale; STAI-S: The State-Trait Anxiety Inventory-State; PROMIS: PROMIS Health Organization anxiety short form; PSQ: Perceived Stress Questionnaire; CES-D: Center for Epidemiological Studies-Depression Scale; n/a: data not reported.

Appendix C

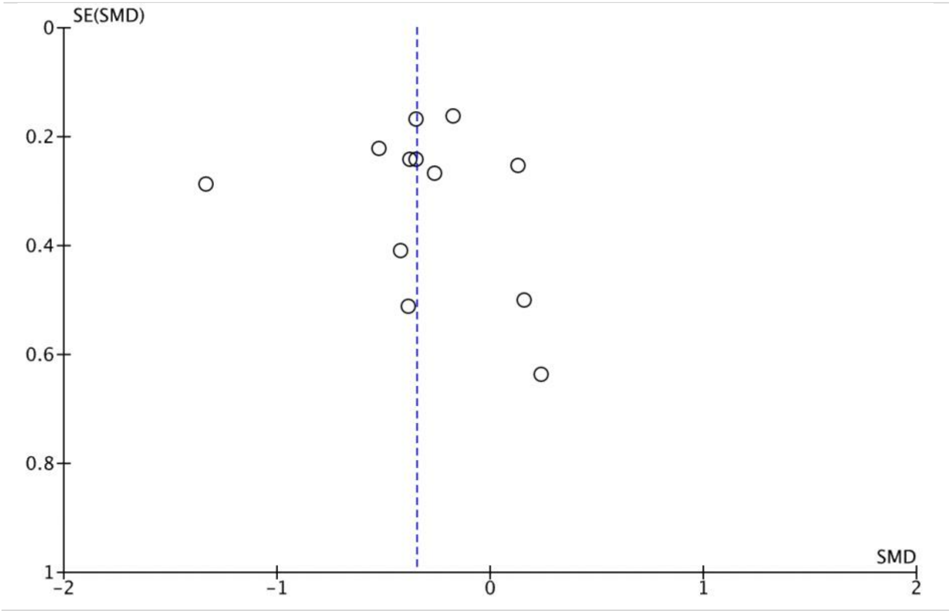

Figure C. Funnel plot comparing breathwork to controls on primary outcome of self-reported/subjective stress at post-intervention. Dotted blue line corresponds to effect estimate and circles represent individual studies. SE = standard error, SMD = standardised mean difference. Figure produced using RevMan v5.4.

Appendix D

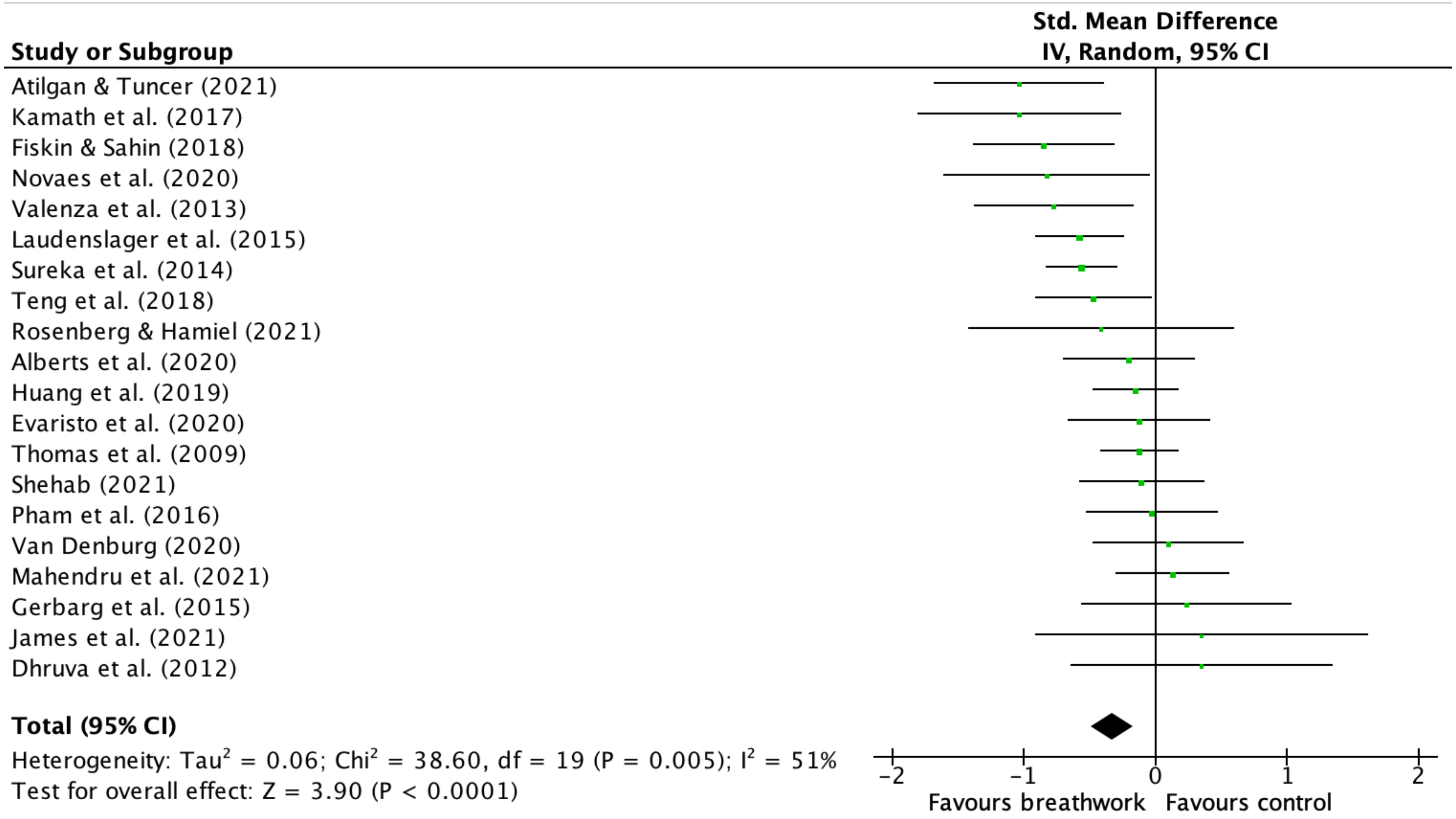

Figure D1. Forest plot comparing breathwork interventions to control groups on secondary outcome of self-reported/subjective anxiety at post-intervention. Green squares and their size represent individual studies and weight of study, respectively. Black lines through squares are 95% CIs and black diamond is the cumulative effect size with 95% CIs. More negative values denote larger effect of breathwork on self-reported/subjective anxiety in comparison to control condition. Figure produced using RevMan v5.4.

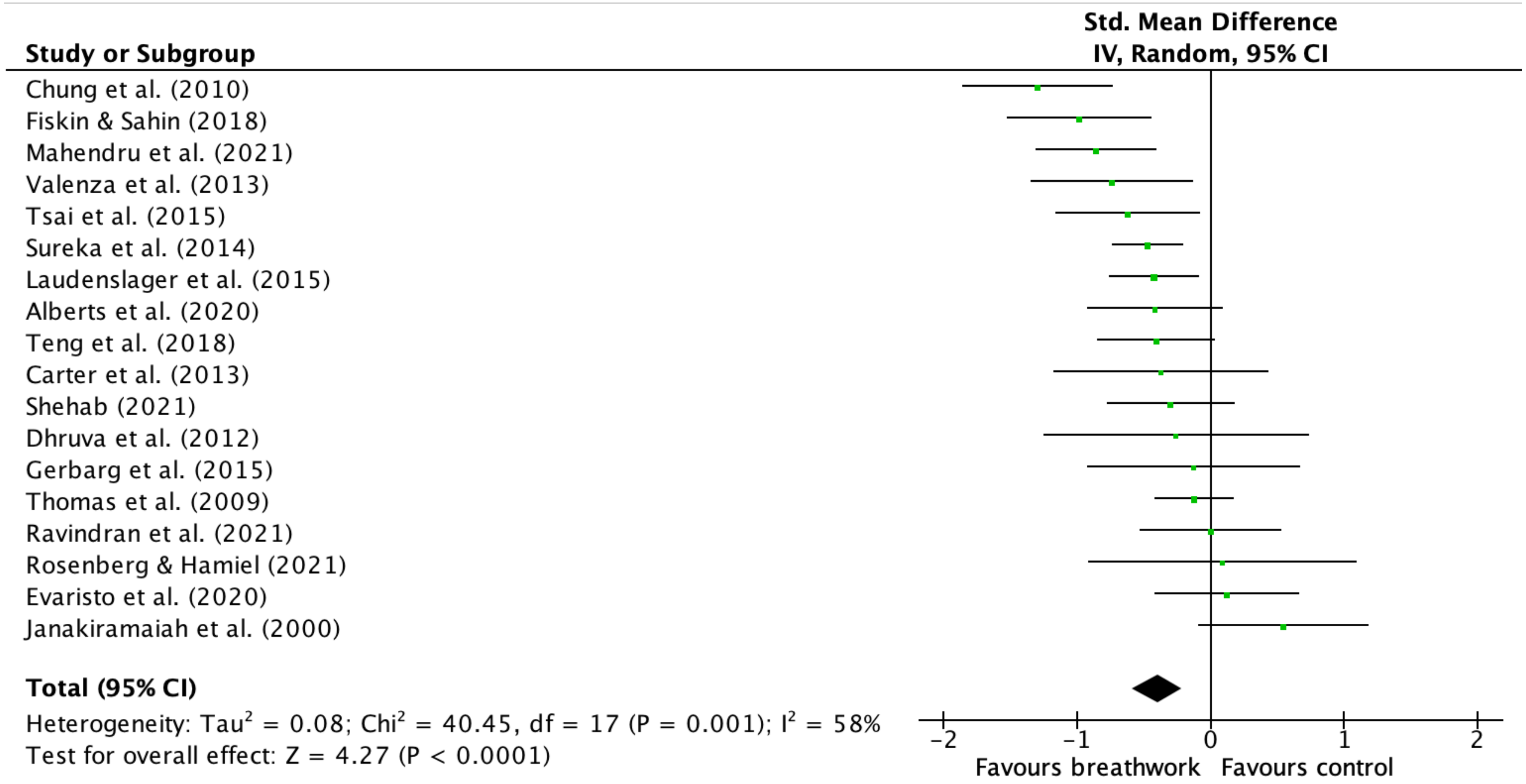

Figure D2. Forest plot comparing breathwork interventions to control groups on secondary outcome of self-reported/subjective depression at post-intervention. Green squares and their size represent individual studies and weight of study, respectively. Black lines through squares are 95% CIs and black diamond is the cumulative effect size with 95% CIs. More negative values denote larger effect of breathwork on self-reported/subjective depressive symptoms in comparison to control condition. Figure produced using RevMan v5.4.
